# CDH1 gene mutation, a challenging surgical topic: Case report and literature review

**DOI:** 10.1016/j.ijscr.2024.109422

**Published:** 2024-02-21

**Authors:** Hani Maalouf, Toufic Saber, Souad Ghattas, Zarouhie Meguerian-Bedoyan, Ziad El Rassi

**Affiliations:** a Department of General Surgery, University of Balamand, Beirut, Lebanon; b Assistant Professor of pathology, Saint Georges Hospital University Medical Center, Beirut, Lebanon; c Head of General Surgery Department, Saint Georges Hospital University Medical Center, Beirut, Lebanon

**Keywords:** CDH1 gene, HDGC, Prophylactic total gastrectomy, E-cadherin, Lobular breast carcinoma, Case report

## Abstract

**Introduction:**

Gastric cancer is one of the top 5 cancers worldwide. Most gastric cancers are classified as sporadic with the exception of around 3 % that are associated with specific syndromes or genes. Hereditary diffuse gastric cancer is a very rare type of gastric cancer, associated with loss of function of a tumor suppressor gene CDH1 which has a high penetrance that can reach 90 % over a lifetime.

**Case presentation:**

Here we present the case of a 31 years old male patient carrying the CDH1 gene who presented for prophylactic total gastrectomy and D1 lymphadenectomy followed by a roux en y esophago-jejunostomy for digestive tract reconstruction. The patient had a preoperative negative gastroscopy for gastric cancer. On final pathology, few 2 mm foci of signet ring cells involving the lamina propria (T1a) were identified.

**Clinical discussion:**

Randomized clinical trial data concerning HDGC is lacking. Individuals who meet the genetic testing criteria developed by the IGCLC, testing should be obtainable from the legal age of consent that range from 16 to 18 years of age. CDH1 is the main gene that is tested. The mainstay treatment of choice for HDGC is total gastrectomy and Roux-en-Y esophago-jejunostomy in asymptomatic patients but should only be undertaken after baseline endoscopy.

**Conclusion:**

Genetic testing for CDH1 should be carried in high-risk populations. Due to its high penetrance, any person carrying the CDH1 gene should be managed by a prophylactic gastrectomy and D1 lymphadenectomy with close follow up for any future breast neoplasm.

## Highlights

• Hereditary diffuse gastric cancer is a very rare type of gastric cancer, associated with loss of function of a tumor suppressor gene CDH1 which has a high penetrance that can reach 90 % over a lifetime.
• Prophylactic total gastrectomy with D1 lymphadenectomy is the only management required post detection of the CDH1 gene.
• Follow up gastroscopy has no place in the management of people carrying this gene because it is operator dependent and false negative gastroscopy can be easily reported due to small foci of cancer. Gastroscopy is only used in a preoperative setting in order to rule out any advance gastric cancer which can affect the surgical management of the case.
• The penetrance of such gene for the development of lobular breast cancer is inferior to the penetrance seen in gastric cancer, thus follow up by yearly mammography and breast ultrasound is an acceptable option post detection of the gene and prophylactic total gastrectomy and lymphadenectomy.

## 1 Introduction

At the present time, gastric cancer is considered to be the fifth most common malignancy and the third leading cause of cancer death universally [1]. Hereditary Diffuse Gastric Cancer (HDGC) comprises 1 to 3 % of all gastric cancers [1,2]. It is an autosomal dominant disease and was first described in an extended New Zealand Māori family in 1998 [3]. HDGC is now estimated to have a worldwide population incidence of around 5 to 10 per 100,000 births [2]. The majority of diagnosed HDGC cases are believed to be due to the inactivating germline mutations in the CDH1 tumor suppressor gene [1].

CDH1 gene codes for E-cadherin that is a calcium-dependent transmembrane glycoprotein that interacts with the extracellular domain to form the adherens junctions [2]. This protein controls embryogenesis and cell maturation, assures epithelial integrity and preserves tissue architecture [1,2]. This mutation is found to be related to HDGC, lobular breast carcinoma (LBC) and cleft lip and or palate and several other diseases [2]. The work has been reported in line with the SCARE criteria [3].

Here we present the case of 31 years old Caucasian male with CDH1 gene mutation and positive family history for HDGC who presented for prophylactic gastrectomy with D1 lymphadenectomy.

## 2 Case report

This is the case of a 31-year-old Caucasian male with a clef lip anomaly followed by repair at a young age and a known CDH1 mutation; presenting for prophylactic total gastrectomy and D1 lymphadenectomy. Due to a strong family history of CDH1 mutation, the patient underwent genetic testing that showed a deleterious loss of function of the corresponding protein. His paternal aunt and uncle died of HDGC and his other paternal aunt had prophylactic total gastrectomy for management of CDH1 gene mutation 5 years ago and now is suffering from lobular breast carcinoma. The patient reports no concerning gastrointestinal symptoms and no systemic symptoms such as fever, night sweats, or weight loss. Vitals were normal upon presentation. Laboratory tests were unremarkable. A gastroscopy reaching the duodenum and a total colonoscopy reaching the ileum were unremarkable and several random gastric biopsies did not show any sign of malignancy. PET scan showed no areas of increased activity suggestive of malignancy or metastatic disease.

A decision for laparoscopic prophylactic total gastrectomy with D1 lymphadenectomy was made. Patient underwent a total gastrectomy with negative frozen section of the distal esophagus and proximal duodenum for gastric mucosa. An End-to-side esophagojejunostomy (EJA) was performed mechanically using an Orvil device inserted in the distal esophageal end, while an EEA stapler was utilized in the jejunal limb. Additionally, Roux-en-Y jejunojejunostomy was performed to prevent bile reflux.75 cm was allocated for the biliary limb and 100 cm for the alimentary limb. D1 lymphadenectomy was done for proper surgical oncological management.

**Fig. 1:**
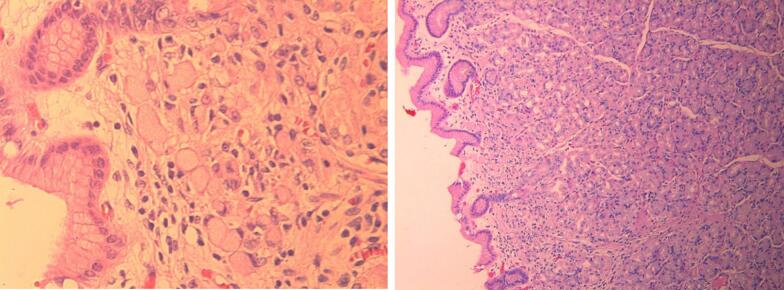
HE: presence of signet ring cells in the superficial lamina propria.

**Fig. 2:**
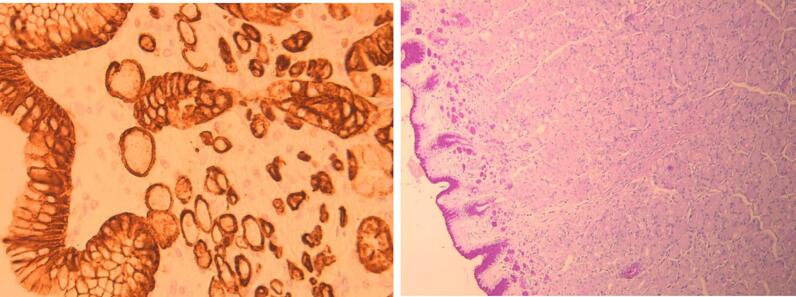
PADS HER2: The PASD positive signet ring cells express Cytokeratin cocktail antigen, confirming their epithelial nature.

Gross examination of the specimen did not show gross polyps or ulcerations. On histopathology, random sections from the gastric wall revealed few foci of up to 2 mm poorly cohesive carcinoma or signet cell carcinoma (WHO classification) or diffuse type gastric adenocarcinoma (Lauren classification) [4]. The signet ring cells (Fig. 1) exhibited PASD and Alcian Blue positive cytoplasmic mucin vacuoles and were expressing strongly the cytokeratin cocktail antigen by immunostaining (Fig. 2), invading the lamina propria, so classified as pT1a. Three microscopic foci were involving the anterior walls of the cardia, body and antrum. HER2/cerbB2 oncogene overexpression was negative by immunohistochemistry. Proximal esophageal and distal duodenal margins were negative for tumor. Twenty-four lymph nodes collected were negative, so classified as pN0.

Post-operative stay was uncomplicated. On day 5 post-operatively, gastrograffin upper gastrointestinal series showed no leak, normal transit, no reflux and no stenosis. The patient was started on liquid diet and was discharged on day 6 post-operatively. Patient was referred to an oncologist for the appropriate follow-up and was advised to undergo yearly breast ultrasound for the associated lobular breast carcinoma with HDGC with no chemo or radio therapy interventions.

## 3 Discussion

HDGC is a rare autosomal dominant cancer disease and is found to be strongly related to diffuse gastric cancer and lobular breast cancer. Mutations in the tumor suppressor gene CDH1 is mainly found to be the cause of this syndrome, and this is true in 40 % of the cases [4,5]. A number of other genes were found to be related to HDGC, namely TP53, RHOA, CTNN1A, and CMTM2 [1,6]. Other causes may be attributed to environmental etiologies. While intestinal type gastric cancer was strongly related to *H. pylori* infection, HDGC is not and is more related Epstein-Barr virus (EBV) infection, though it is still not clearly understood [1]. Other factors such as tobacco use, diet, alcohol, or certain lifestyle aspects also appears to be related to the development of DGC and merit further research [1].

The management decision was made during a multidisciplinary team (MDT) meeting involving oncologists, pathologists, gastroenterologists, surgeons, and radiologists. Genetic counseling is crucial during this process. He underwent genetic testing preoperatively, which played a significant role in the decision to proceed with surgery. In the future, genetic testing should be routinely included in the management of CDH1 patients as part of the preoperative assessment.

Due to the fairly low incidence of HDGC, randomized clinical trial data concerning HDGC is lacking. Subsequently, the diagnosis and management of this disease relies on recommendations developed by consensus expert evidence and opinion and on observational studies [2]. The International Gastric Cancer Linkage Consortium (IGCLC) has released an update in August 2020 on the guidelines for HDGC covering genetic testing, endoscopic and histological surveillance and surgical management of this rare developing disease [2].

In individuals who meet the genetic testing criteria developed by the IGCLC, testing should be obtainable from the legal age of consent that range from 16 to 18 years of age. Younger family members should be offered testing based on family history [7]. CDH1 is the main gene that is tested. A-catenin (CTNNA1), a second adherens junction protein other than CDH1, is also found in a small minority of HDGC cases. Mutations of which should also be tested [8]. Criteria are divided into family and individual criteria. Family criteria include: more than 2 cases of DGC or LBC in family members or more than 1 case of DGC and more than 1 case of LBC in a different family member. Individual criteria include but are not limited to a DGC at an age younger than 50, DGC at any age in those of Māori ethnicity, DGC in those with a personal or family history (1st degree) of cleft lip/cleft palate, history of DGC and LBC, both diagnosed at an age younger than 70, and Gastric in situ signet ring cells in those younger than 50 years of age [2].

Cleft lip and or palate was also found to be associated to CHD1 mutation and HDGC disease, hence why it is included in the genetic testing criteria [2]. It was found that CDH1 is highly expressed at weeks 4 and 5 of the development in the frontonasal prominence, and at week 6 in the lateral and medial nasal prominences of embryos and is consequently expressed during the critical stages of lip and palate formation [9].

Our patient fulfilled the criteria for genetic testing since he had a cleft lip upon birth and had a strong family history of HDGC with two paternal aunts affected, one who survived following total gastrectomy and the other one that died, and an uncle who was also affected and is diseased.

The role of endoscopy in the diagnosis and surveillance of HDGC is still not well understood. Finding Signet ring cell carcinoma (SRCC) lesions in endoscopic biopsies is uncertain, particularly since these superficial SRCC foci can have a very indolent behavior and sometimes can only be caught in 40 to 60 % of the cases [9]. According to the IGCLC, endoscopy should be done in highly trained centers for HDGC to increase the chances of catching the cancer [2]. The probability of having at least one SRCC lesion in the total gastrectomy specimen from a CDH1 mutation carrier is 95 % [10]. This shows the high penetrance of this gene and hence, regardless of the result of the endoscopy, prophylactic total gastrectomy (PTG) should be offered to all CDH1 positive patients [1,2]. Surveillance instead of a PTG can be considered in certain cases including patient's refusal of surgery, pathogenic variant carriers, patients with affected family members from ‘HDGC-like’. In the case of not performing a surgery, the patient should be well educated for the risk of developing the cancer and the limitations of this decision [2].

The upper age limit for surveillance endoscopy is around 70 years of age and this depends on the fitness for gastrectomy. Endoscopies done for surveillance should include both targeted and random biopsies. Random biopsies should reach the number of 28 to 30 taken from all over the stomach [2].

The mainstay treatment of choice for HDGC is total gastrectomy and Roux-en-Y esophago-jejunostomy in asymptomatic patients but should only be undertaken after baseline endoscopy [1,2]. Intraoperative confirmation of esophageal proximal margin and duodenal distal margin should be insured. Perigastric lymph node metastases are exceptionally uncommon in patients undergoing PTGs. Therefore, an extended D2 lymphadenectomy is not required and is avoided to minimize postoperative morbidity and a D1 lymph node dissection is more recommended as missing a focus of cancer in the setting of prophylactic gastrectomy is not uncommon [1,2]. A D2 dissection will be granted for T1 and node positive cases, hence the role of pre-operative need of gastroscopy [1]. If the gastric cancer invades the duodenum, D2 dissection is extended to involve retro-pancreatic lymph nodes and this dissection is defined as D2+ [12]. Intraoperatively, more than15 lymph nodes are recommended to be removed and examined for accurate staging [13].

Our patient had pre-operative gastroscopy done with negative gastric tumor on pathology from the biopsy sections taken and negative lymph nodes, so a decision for PTG was undertaken with D1 level lymphadenectomy. Hansford et al. [14] estimated a cumulative risk of 70 % for developing HDGC by age the age of 80 in families who had CDH1 mutation. This penetrance varies according to lifestyle factors and in between families and hence, family history should be considered when studying an individual's risk. Looking at the high penetrance of this disease and to the high percentage of the positive HDGC that can reach 95 % [11] on histopathologic specimen from PTG patients, the safely endoscopic surveillance provided by IGCLC is questioned and can be considered hazardous since it can delay diagnosis and treatment of this condition. And the findings of cancer in the final pathology in our case report, confirms that endoscopy can be misleading and falsely negative and the need for surgery will be eventually inevitable. And in this case, endoscopy can only help in the lymphadenectomy level decision.

The role systemic therapy in the management of HDGC is still not well understood or studied. What is already acknowledged, is that there is decreased responsiveness to chemotherapy and chemoradiation in the treatment of HDGC. In the absence of metastasis, a publication of the MAGIC trial and more recently FLOT4-AIO trial showed the advantage of peri-operative chemo-radiation in the treatment of HDGC. Hence, still further studies should be conducted to reach an efficient management of this rare emerging disease [15].

Complications of prophylactic total gastrectomy include bile reflux, which is the most common, resulting in symptoms such as abdominal pain, heartburn, and nausea, as well as difficulty swallowing and challenges in absorbing certain nutrients. Other complications include venous thromboembolism, anastomotic leaks, and intra-abdominal abscesses. However, the overall benefits of the procedure outweigh these risks, particularly in preventing the development of clinically relevant cancer [16].

## 4 Conclusion

With the advance of molecular testing, the incidence of detecting CDH1 will be on the high rise especially in patients with family history of cancers. This gene has a high penetrance and is associated with the development of diffuse gastric cancer and lobular breast cancer. Prophylactic total gastrectomy with D1 lymphadenectomy is the only management required post detection of the CDH1 gene. Follow up gastroscopy has no place in the management of people carrying this gene because it is operator dependent and false negative gastroscopy can be easily reported due to small foci of cancer. Instead, gastroscopy is only used in a preoperative setting in order to rule out any advance gastric cancer which can affect the surgical management of the case.

## Consent for publication

Written informed consent was obtained from the patient for publication and any accompanying images. A copy of the written consent is available for review by the Editor-in-Chief of this journal on request.

## Provenance and peer review

Not commissioned, externally peer reviewed. This work has been reported in line with the SCARE criteria: Sohrabi C, Mathew G, Maria N, Kerwan A, Franchi T, Agha RA. The SCARE 2023 guideline: updating consensus Surgical Case Report (SCARE) guidelines. Int J Surg Lond Engl. 2023;109(5):1136.

## Ethical approval

Case report approved for publishing by ethical committee at Saint George hospital University Medical Center, and Head of General Surgery division, Beirut, Lebanon, 2023.

## Funding

Faculty of Medicine and Medical Sciences, 10.13039/501100007303University of Balamand, Lebanon.

## Author contribution

Hani Maalouf MD (First Author), Toufic Saber MD (Co-Author), Souad Ghattas MD (Co-Author), Zarouhie Meguerian-Bedoyan MD (Co-Author), Ziad El Rassi MD (Corresponding Author).

## Guarantor

Hani Maalouf.

## Declaration of competing interest

No competing interests were declared by the authors.
